# Modern Sunni-Shia conflicts and their neglected tropical diseases

**DOI:** 10.1371/journal.pntd.0006008

**Published:** 2018-02-15

**Authors:** Peter J. Hotez

**Affiliations:** 1 Texas Children’s Hospital Center for Vaccine Development, Departments of Pediatrics and Molecular Virology and Microbiology, National School of Tropical Medicine, Baylor College of Medicine, Houston, Texas, United States of America; 2 James A Baker III Institute for Public Policy, Rice University, Houston, Texas, United States of America; 3 Department of Biology, Baylor University, Waco, Texas, United States of America; 4 Scowcroft Institute of International Affairs, Bush School of Government and Public Service, Texas A&M University, College Station, Texas, United States of America; National Institutes of Health, UNITED STATES

The political scientist and Middle East and Islamic specialist Vali Nasr has written extensively about the current wars and conflicts in the Middle East, attributing many, if not most, of these activities to recent escalations in ancient Shia-Sunni disputes [1]. Nasr writes: “The Middle East today is more vulnerable to instability and extremism than at any time since Iran’s Islamic revolution…” and goes on to suggest that the current wars in Syria, Iraq, and Yemen may represent an Iranian-Saudi rivalry by proxy, noting that such rivalries intensified beginning in 1979 [1]. Ultimately, he points out that “[t]he Middle East is bound to go through—indeed, is now going through—a period of violence as the old order gives place to a new one and Shias and Sunnis adjust to the new realities” [1].

**Fig 1:**
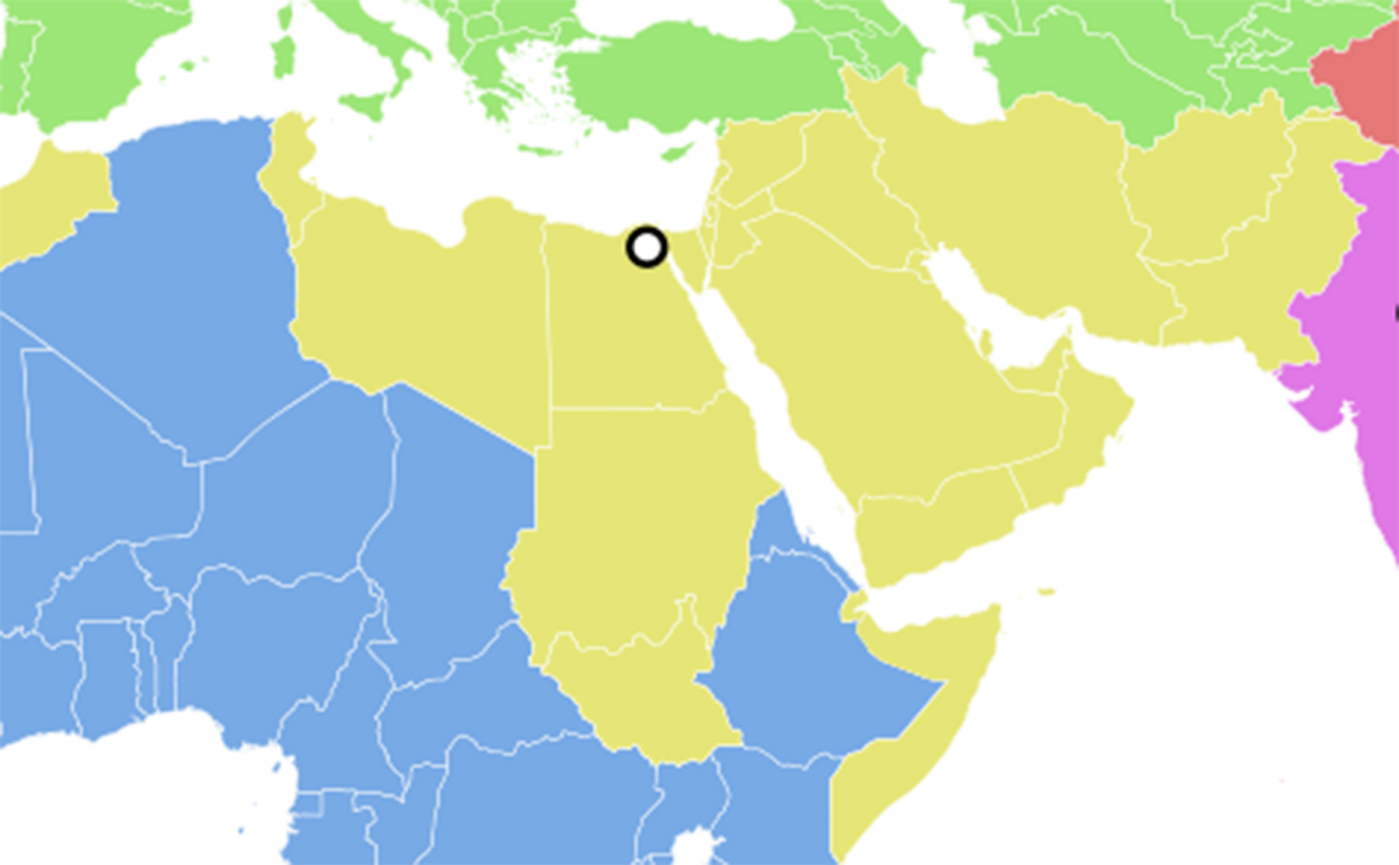
The World Health Organization’s Eastern Mediterranean Region (EMR). The circle shows the location of headquarters in Cairo, Egypt. Modified from https://en.wikipedia.org/wiki/WHO_regions#/media/File:World_Health_Organisation_regional_offices.svg.

In the meantime, we are only now beginning to fully understand the global health impact of the current geopolitical situation in the Middle East and surrounding areas. The Global Burden of Disease Study (GBD) 2015 has recently undertaken an extensive analysis of health in the World Health Organization’s Eastern Mediterranean Region (EMR), an area that includes the countries of the Middle East and North Africa, as well as Afghanistan and Pakistan in Central Asia, and Somalia and Sudan in East Africa, which are nations also beset by conflict (Fig 1) [2–4].

The GBD 2015 EMR Collaborators led by Dr. Ali Mokdad indeed find that the factors of war, mass human displacement, and political instability have blocked improvements in health for the region, with resultant reductions in life expectancies in Egypt, Yemen, Libya, and Syria, as well as a rise in mental health and drug-use disorders [2]. They also determined that war and law enforcement now represent the top-ranked contributors to poor health leading to death for adolescents and young adults [3].

We know that war, political instability, and human migrations are also important factors that promote the emergence of infectious diseases, especially vector-borne or zoonotic neglected tropical diseases (NTDs) [5]. Such forces explain the emergence or reemergence of sleeping sickness in Central Africa and kala-azar in East Africa during the 1990s and Ebola in West Africa in 2014 [5]. Another important factor promoting vector-borne and zoonotic NTDs is climate change [5], and the EMR has been severely affected in this regard, with record-setting hot temperatures and shifting rainfall patterns in recent years [6].

According to the GBD 2015, however, overall the disease burden from infectious diseases in the EMR has declined significantly between the years 1990 and 2015 [4]. For example, in 1990 the EMR’s leading causes of disability-adjusted life years (DALYs) were lower respiratory infections and diarrheal disease, while measles ranked 7th, tuberculosis ranked 15th, and tetanus ranked 16th [4]. Fast forward to 2015, and we find that the DALYs from diarrheal disease have dropped by 25.6% so that it now ranks 8th, while tuberculosis has dropped to 22nd, and measles and tetanus no longer rank in the top 30 leading causes of DALYs [4]. In their place, noncommunicable diseases (NCDs) have increased substantially [4].

But when it comes to looking specifically at the NTDs in the war zones, the overall findings for the EMR’s shifting balances between NCDs and infectious diseases may mask some ominous trends in terms of the emergence or reemergence of several key diseases (Box 1).

**Box 1. Emerging or reemerging neglected tropical diseases (NTDs) in the Eastern Mediterranean Region (EMR)**

- Cutaneous leishmaniasis
- Visceral leishmaniasis
- Cholera
- Dengue
- Rift Valley fever
- Alkhurma hemorrhagic fever
- Crimean Congo hemorrhagic fever

One important example is cutaneous leishmaniasis. According to the GBD 2015, cutaneous leishmaniasis does not rank among the 30 leading causes of DALYs in the EMR [4], but alternative estimates suggest that the DALYs from this condition may be severely underestimated because patients with inactive disease who are left with permanent scars are not fully considered in GBD estimates, nor are the psychosocial consequences of facial scars [7]. The GBD 2013 reports that the global prevalence of cutaneous leishmaniasis has increased 174% since 1990, with much of that increase attributed to a sharp uptick in the number of cases in the EMR [8, 9]. Indeed, the 5 leading countries in terms of cutaneous leishmaniasis prevalence rates are all in the EMR, led by Afghanistan [8, 9].

Precise incidence rates and the exact number of new cases of cutaneous leishmaniasis in the conflict areas of Afghanistan, Syria, Iraq, Libya, and Yemen are difficult to assess because of the lack of active surveillance activities amid the collapsed health systems in these nations, but initial assessments indicate high rates of the disease [10–13]. Compared to the years before the Syrian conflict, for instance, the numbers of cutaneous leishmaniasis cases are thought to have since increased 2–5-fold to over 100,000 cases in 2014 alone, due to the stoppage of vector control activities and the creation of new sand fly breeding sites amid the bombed-out rubble in cities such as Aleppo [13]. Subsequently, some indoor residual spraying has resumed so that the expected numbers of new cases may not be as high [13]. However, cutaneous leishmaniasis remains hyperendemic in Syria, and the reemergence of visceral leishmaniasis has now also been noted [13].

Because refugees fleeing conflict areas of Syria also exhibit high prevalence of rates of cutaneous leishmaniasis, they may introduce the disease in surrounding countries where sand flies are found, especially in Turkey, Jordan, and Lebanon [11, 12]. Also worrisome is the worsening situation in Yemen, where the conflict there could promote the reemergence of cutaneous leishmaniasis.

Yemen is also the site of the EMR’s other major NTD outbreak, now resulting in a massive increase in cholera cases. According to the World Health Organization, the total number of cases now exceeds 500,000, with almost 2,000 deaths, such that Yemen currently represents the world’s largest cholera epidemic [14]. The health system of Yemen is believed to be collapsing, and similar to the cutaneous leishmaniasis situation in Syria, there are shortages of medicines and supplies [14]. It’s interesting to note that despite similar circumstances and healthcare collapses in Syria, so far there have not been major outbreaks of cholera or other diarrheal diseases [13].

The EMR region continues to be at risk for additional outbreaks of NTDs. It has been noted that dengue and other arbovirus infections may be reemerging and causing large outbreaks in Pakistan and on the Arabian Peninsula and East Africa, especially in areas that border the Red Sea [15]. Egypt also experienced a dengue outbreak in 2015 [15]. There is an urgent need to better understand the drivers for dengue emergence and to determine the relative contributions of conflict, climate change, and human migrations and commerce [15]. There are also several other emerging NTDs of concern, including Rift Valley fever previously introduced into the Arabian Peninsula from Kenya, and several other viral hemorrhagic fevers (HFs), including Alkhurma HF and Crimean-Congo HF [16, 17].

Over the last few years, the EMR has emerged as the newest important “hot zone” for emerging and reemerging NTDs. We have also seen important vaccine-preventable childhood diseases reappear, such as measles and polio [18], in addition to the Middle East Respiratory Syndrome (MERS) coronavirus [19, 20]. Tuberculosis is also a major contributor to the premature death of young adults [4], but the extent to which this finding represents a new situation remains unclear. Overall, most investigators attribute a rise in tuberculosis rates to the ongoing hostilities [11].

While war and political instability in the EMR have become a major driver for NTDs and other poverty-related neglected diseases, we may be witnessing how conflict pairs with other forces such as climate change, shifting patterns of poverty, and human migrations linked to the Hajj [19, 20]. A major concern is that the NTDs now rising in the conflict areas are spilling into other EMR areas and potentially could ignite a pandemic. In 2014, we saw how Ebola arose in the setting of collapsed health systems due to the atrocities in Guinea, Liberia, and Sierra Leone and then threatened to overtake much of West Africa and beyond. We may be witnessing a similar set of forces and drivers in the EMR [19, 20].

Professor Nasr has pointed out that “[t]he Shia-Sunni conflict is at once a struggle for the soul of Islam—a great war of competing theologies and conceptions of sacred history—and a manifestation of the kind of tribal wars of ethnicities and identities….with which humanity has become wearily familiar” [1]. But almost certainly the Sunni-Shia divide is not the only driver of political instability leading to disease emergence. Today, the world’s Islamic nations belonging to the Organization of Islamic Cooperation (OIC) account for a high percentage of the global burden of NTDs [21]. Potentially one or more of these diseases could soon consume the EMR and possibly other parts of the Islamic world. We therefore need to identify mechanisms to isolate or control the NTDs now emerging from the conflict zones and promote better cooperation between the warring factions for this purpose. Ultimately, the Muslim world has been rendered highly vulnerable to NTDs, which will further destabilize OIC countries and promote poverty.

Ultimately, we can look to science and vaccine diplomacy to promote disease control activities across the EMR and to build new interventions for preventing the spread of the new and emerging NTDs [19, 20].

There are several possible approaches to consider on this front. First, greater cooperation between the OIC nations could increase access to essential medicines for mass treatment of the NTDs, including intestinal helminth infections, schistosomiasis, lymphatic filariasis, onchocerciasis, and trachoma. Today, in some of the African Sahelian OIC nations, only a small percentage of the at-risk populations (including children) benefit from regular and periodic mass drug administration. This problem could be addressed through increased financial support and technical cooperation among OIC nations.

In addition, there is an urgent need to create new vaccines for some of the NTDs and other poverty-related neglected diseases arising out of the conflict nations highlighted above. We need new vaccines for leishmaniasis, coronavirus infections, viral HFs, and other diseases. In terms of nations adjacent to Middle East conflict zones, currently both Saudi Arabia and Iran, as well as other Gulf Cooperation Council (GCC) nations, have substantial capacities for vaccine biotechnology. We need to do better tapping into those strengths. In my role during 2015 and 2016 as United States science envoy for the State Department and White House, I worked to expand collaborations between the US and Saudi Arabia, now leading to joint scientific activities in the area of vaccine development. But such initiatives in the area of vaccine diplomacy need to be expanded. Could US-Saudi vaccine diplomacy extend to other nations? It would be worthwhile to also explore the inclusion of other nations in the Middle East, North Africa, and Central Asia, particularly those that do not have historical connections to Saudi Arabia. In so doing, vaccine diplomacy could become a key 21st-century theme to address regional NTDs arising out of conflict and to promote international cooperation in the region and among the OIC nations.
